# Maternal and Child Acceptability of a Proposed Guided Imagery Therapy Mobile App Designed to Treat Functional Abdominal Pain Disorders in Children: Mixed-Methods Predevelopment Formative Research

**DOI:** 10.2196/pediatrics.8535

**Published:** 2018-06-29

**Authors:** John M Hollier, Adetola O Vaughan, Yan Liu, Miranda AL van Tilburg, Robert J Shulman, Debbe I Thompson

**Affiliations:** ^1^ Section of Gastroenterology, Hepatology, and Nutrition Department of Pediatrics Baylor College of Medicine Houston, TX United States; ^2^ Texas Children's Hospital Houston, TX United States; ^3^ Department of Medicine Baylor College of Medicine Houston, TX United States; ^4^ College of Pharmacy & Health Sciences Department of Clinical Research Campbell University Biues Creek, NC United States; ^5^ Department of Medicine University of North Carolina Chapel Hill, NC United States; ^6^ School of Social Work University of Washington Seattle, WA United States; ^7^ Children's Nutrition Research Center Agricultural Research Service United States Department of Agriculture Houston, TX United States; ^8^ Department of Pediatrics Baylor College of Medicine Houston, TX United States

**Keywords:** functional abdominal pain disorders, guided imagery therapy, mixed methods, mobile applications, pediatrics, parents, Technology Acceptance Model, imagery (psychotherapy)

## Abstract

**Background:**

Functional abdominal pain disorders are chronic abdominal pain conditions, which affect up to 20% of children worldwide. Of the various functional abdominal pain disorder treatment modalities, psychological therapies such as guided imagery therapy appear most effective. However, there are significant barriers to receiving psychological therapies, including access to trained therapists. Alternatively, remotely delivered psychological therapies for functional abdominal pain disorders have been efficacious.

**Objective:**

The objective of our study was to assess acceptability of a proposed guided imagery therapy app designed to treat functional abdominal pain disorders through remote delivery of prerecorded audio sessions and to evaluate user preferences for using such an app.

**Methods:**

Using a mixed-methods approach, we conducted a predevelopment formative study among children aged 7 to 12 years with a functional abdominal pain disorder and their parents. The parents completed our modified Technology Acceptance Model (TAM) questionnaire, which quantified behavioral intention and related factors for using a guided imagery therapy app. Dyads participated in separate in-person semistructured interviews to assess their attitudes toward and preferences for a guided imagery therapy app. Questionnaire and interview findings were collected concurrently, analyzed separately, and then integrated through methods triangulation.

**Results:**

Among the 15 participating parent-child dyads, 5 (33%) children were Hispanic and 11 (73%) had irritable bowel syndrome. They had diverse socioeconomic status. All parent participants were mothers. The TAM questionnaire indicated that mothers scored favorably on behavioral intention to use a guided imagery therapy app (mean score 12.0, SD 2.6, possible range 3-15). Scores for the TAM factors perceived usefulness, perceived ease of use, hedonic motivation, compatibility, and habit also were favorable. Maternal interviews confirmed positive attitudes toward the proposed app. They advocated a visual component to hold their child’s attention during the guided imagery therapy sessions; recommended incorporating background sounds into the sessions; favored session reminder notifications from the app; and thought the best time for their child to listen to the sessions would be in the evening or before bed. The child interviews also confirmed positive attitudes toward the proposed app. They suggested guided imagery therapy session topics such as sports and adventures; listening to sessions in their bedroom; and the need for parental supervision to install the app on their mobile device. Integration of the quantitative and qualitative methods findings complimented one another on acceptability. The favorable behavioral intention TAM score aligned well with expressed positive maternal and child attitudes toward the app and can be explained by the desire to avoid medications. The questionnaire and interviews also confirmed therapeutic benefit as an intrinsic motivator to promote routine use.

**Conclusions:**

A guided imagery therapy app designed to treat pediatric patients with functional abdominal pain disorders appears to be acceptable to both mothers and children. Incorporating parent and child preferences into a guided imagery therapy app could promote therapeutic compliance and increase access to optimal care.

## Introduction

Functional abdominal pain disorders (FAPDs; eg, irritable bowel syndrome, functional abdominal pain, and functional dyspepsia) are chronic abdominal pain conditions that cannot be ascribed to a particular biochemical or anatomical abnormality [1]. These disorders affect about 20% of school-aged children and adolescents worldwide and are associated with psychological distress, such as anxiety and depression [2-10]. Affected children often have a decreased quality of life to a greater degree than those with organic diseases such as gastroesophageal reflux disease and inflammatory bowel disease [6,11,12].

Mainstay treatment options for FAPDs include conservative medications, alternative medications, diet modification, and psychological therapy [1,13,14]. Recently published Cochrane reviews support psychological therapies over medications for treating FAPDs in children [15,16]. Cognitive behavioral therapies and related therapies such as guided imagery therapy (GIT) appear to be the most efficacious psychological treatments for pediatric patients. GIT is based on a cognitive-behavioral framework that uses the imagination to reduce anxiety and stress [17,18]. A traditional in-person GIT session involves an alert person relaxing and following a practitioner’s audio commands to imagine various sensory images, which in turn serve as a mental representation of a concept such as abdominal pain. Afterward, the patient is able to modulate their pain using this mindfulness technique [19-21]. The three principles of GIT are mental imagery, an altered state as induced by trance, and a locus of control that enables a person to control their situation [22]. This therapy is often used for psychological and chronic pain disorders such as anxiety and fibromyalgia [23-25].

Access to psychological approaches such as GIT is hindered by various systemic barriers such as lack of trained practitioners, limited insurance coverage for mental health services, and the need for repeated visits [26,27]. To overcome these barriers, researchers have studied whether psychological therapies delivered remotely can decrease abdominal pain symptoms related to FAPDs. Both gut-directed hypnotherapy and GIT delivered via compact disc have been shown to decrease pain symptoms in children with FAPDs [28-30]. Thus, psychological therapies delivered remotely have the potential to overcome these systemic barriers affecting access to optimal care for affected children.

The World Health Organization defines mHealth as “medical and public health practice supported by mobile devices” [31]. Given the ubiquity of electronic mobile devices in our contemporary society, translating the remote delivery of prerecorded psychological therapy sessions to a mobile app would create a ubiquitous mHealth clinical tool that could improve health outcomes of many children with FAPDs [32,33]. This mobile therapy approach has been used successfully for adult psychological disorders, including anxiety, depression, and mood disorders [34,35]. Furthermore, mobile apps for anxiety and depression have been developed for children [36,37]. Considering the preliminary success of remotely delivered GIT for FAPDs, a GIT mobile app could transform our paradigm for treating children with FAPDs if our target population would find this clinical tool acceptable.

Technology acceptance of a clinical tool depends on several factors, including satisfaction and perceived appropriateness [38]. The literature and experts at the Institute of Medicine and Robert Wood Johnson Foundation in the United States, and the National Health Service in the United Kingdom, recommend integrating the preferences of stakeholders, including the target population, into the mobile health app and other electronic interventions and recommend conducting formative research to capture this information prior to app design and development [39-41]. These studies and organizations also support the utility of formative research to capture stakeholders’ preferences for an electronic mHealth clinical intervention. Unfortunately, the vast majority of heath apps, including psychological mobile apps, have not followed these recommendations and, indeed, commonly lack evidence of efficacy [34-37]. Thus, our long-term goal is develop an efficacious GIT mobile app following the guidelines outlined above.

Modi et al [42] conceptualized a framework for self-management in the pediatric population and outlined multiple behavioral influences on pediatric health, including individual, family, and community influences, which is similarly summarized in the social ecological model [42,43]. Furthermore, pediatric obesity studies have shown that parents are key agents of change for weight loss and behavior change to promote weight loss [44]. Based on these studies, our formative research study also aimed to capture important environmental and family influences that could affect the sustained use of a GIT mobile app designed for children with FAPDs.

Given the above background, the purpose of our formative study was to assess the acceptability and appropriateness of using a GIT mobile app among children with FAPDs and their parents. We also evaluated whether specific preferences or barriers to use would affect the therapeutic compliance of such a clinical tool.

## Methods

### Participants

This study recruited pediatric patients aged 7 to 12 years with a FAPD and their parents through volunteer sampling. We selected this age range because these children are old enough to have thoughts that are developmentally logical and organized enough to participate in therapy, but they have not yet reached the stage of autonomy seen in adolescents, who characteristically have poor self-management health behaviors [42,45]. We excluded participants who had other comorbidities associated with chronic abdominal pain, including abdominal surgery and other diagnoses (eg, diabetes mellitus, cystic fibrosis) [46,47].

Patients were recruited in three ways from a large metropolitan ambulatory health care system in Houston, Texas, USA. The first method entailed recruiting patients from another noninterventional clinical study conducted within our research group. The second method involved posting flyers in 52 ambulatory primary care clinics affiliated with a tertiary pediatric health care system in which interested families could contact our research team for possible enrollment. The final method involved sending letters directly to families who had recently visited one of our primary care clinics for abdominal pain, with the approval of their treating primary care physician. If the families did not respond to the letters, we attempted to contact them by phone. This study was approved by the Baylor College of Medicine Institutional Review Board (H-37308).

Interested families completed the pediatric Rome III questionnaire to ensure that the patient met the criteria for a FAPD. The Rome III questionnaire is a validated instrument that assesses abdominal pain and stooling patterns in children and determines whether a FAPD is present and, if so, its respective classification (eg, irritable bowel syndrome) [48,49]. Potential participants also agreed to have the child’s medical record screened for additional inclusion and exclusion criteria. Once a parent agreed to participate, written parental informed consent of study participation and child assent were obtained at the start of the research visit.

### Data Collection

#### Mixed-Methods Study Design

This mixed-methods study used a complementary design consisting of a parental quantitative questionnaire and separate parent and child in-person interviews [50,51]. Both quantitative and qualitative phases contributed equally to the final study results [51]. Experts cite the purpose of a mixed-methods approach as 2-fold: to verify that recorded data from two different methods can be corroborated, and that the overall data captured are robust and comprehensive [52,53]. We used methods triangulation of both a quantitative questionnaire and qualitative interviews for both of these purposes [54]. For this study, we integrated the results of our quantitative questionnaire and qualitative interview findings and assessed whether the results were complimentary or discordant to one another, and also determined whether our qualitative interviews could further explain the quantitative questionnaire findings.

#### Demographic and Clinical Characteristics Questionnaires

Parents completed self-report questionnaires that assessed parent and child demographic characteristics and household members’ access to electronic devices; they also completed the pediatric Rome III questionnaire, which captured the affected child’s FAPD [47]. We obtained the annual median household income based on the family’s zip code [47,55]; using 2015 salary estimates [55], we averaged the sample’s median annual household income as a proxy measure for socioeconomic status [56].

#### Guided Imagery Therapy Demonstration

Parent and child dyads were separated for semistructured interviews. The first portion of each interview involved explaining that the research team was interested in using a mobile app that played GIT sessions on an electronic mobile device to treat children with FAPDs. The interviewer then showed the participant an electronic tablet with mobile app icons on its home screen to demonstrate the platform envisioned by the research team. Afterward, the interviewer played a 2-minute GIT session audio excerpt on the electronic tablet, which was derived from a track previously demonstrated to be efficacious in a pediatric clinical study (excerpt provided by MALvT) [28].

#### Modified Technology Acceptance Model Questionnaire for a Guided Imagery Therapy Mobile App

After the audio demonstration, parents were asked to complete a survey containing questions related to their child’s demographics, family technology use at home, and a modified version of the Technology Acceptance Model (TAM) questionnaire. The TAM was developed based on the theory of reasoned action/theory of planned behavior and is tailored to assess users’ acceptance and use of modern technologies [57,58]. Central tenants of these models are the multiple factors that influence behavioral intention to do a specific task. The Institute of Medicine defines behavioral intention as the “subjective probability that he or she will engage in a given behavior” [59]. The theory of reasoned action/theory of planned behavior posit that the behavioral intention to engage in a particular health behavior is highly associated with an actual health behavior [57,58]. The original TAM model included the domains of perceived usefulness (the degree to which a person believes that using a technology would enhance his or her performance), perceived ease of use (the degree to which a person believes that using a technology would be free of effort), and attitude (how much a person likes the object of thought and their related beliefs about the thought), all acting on behavioral intention to use a technology [57,60,61]. Over time, the model expanded to include the domains of compatibility (the extent in which the technology fits a person’s experiences or activities), habit (a perceived link between a goal and a specific behavior), social influence (the person’s perception of a referent other’s opinion about the person’s use of a technology), and hedonic motivation (the intrinsic drive to use a technology) [62-65].

**Figure 1:**
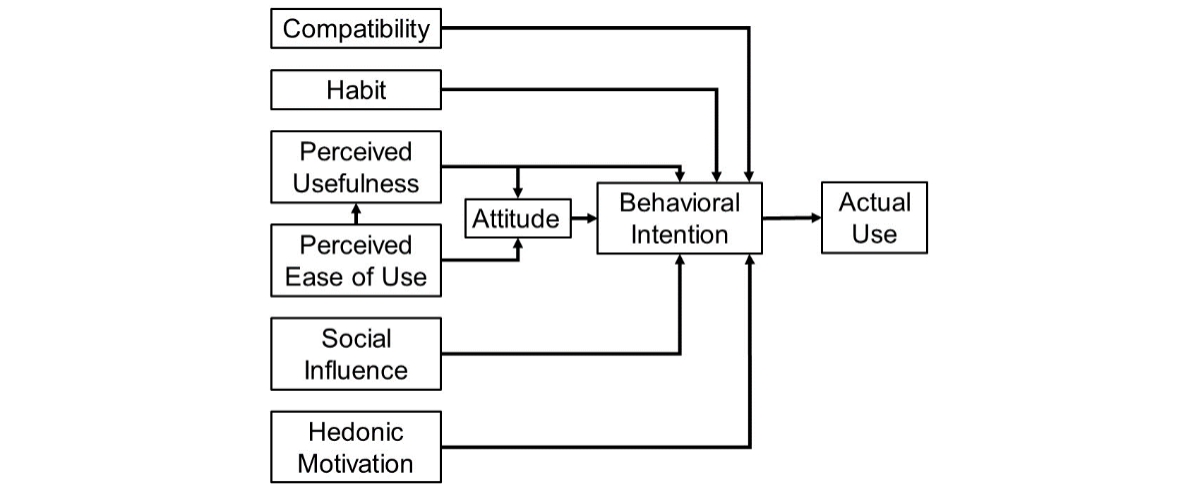
Modified Technology Acceptance Model.

For this study, we adapted the original TAM questionnaire to assess how parents in this sample rated various TAM factors and behavioral intention to use a proposed GIT mobile app to treat their child’s FAPD. Our questionnaire was based on questionnaires used in 5 other TAM studies [66-70]. The final modified TAM questionnaire consisted of 33 questions that assessed the factors of perceived usefulness, perceived ease of use, attitude, compatibility, habit, and behavioral intention to use a GIT mobile app for FAPDs. Questionnaire items were scored on a 5-point Likert scale: 1=strongly disagree, 2=disagree, 3=neutral, 4=agree, and 5=strongly agree (Multimedia Appendix 1). Figure 1 shows an adapted version of the modified TAM summarizing the factors that influence people’s use of a modern technology [66-70].

#### Interviews

Separate semistructured interviews were conducted for parents after the surveys and after the GIT demonstration for the children. Interviews were conducted by trained interviewers following a script (see Multimedia Appendix 2 for the parent interview script and Multimedia Appendix 3 for the child interview script). Script questions were guided by the social ecological model and the theory of planned behavior/theory of reasoned action [58,71]. The parent script contained 12 questions, while the child script consisted of 15 questions. Although the scripts contained similar questions, the child script used simpler terminology and captured less information regarding app logistics for use; the different script questions also reflected the different roles that parents (agent of change) and children (user) would be expected to assume. Probes and prompts were used as needed to expand and clarify responses. Interviews were designed to last no longer than 1 hour. All interviews were digitally recorded.

### Data Analysis

#### Modified Technology Acceptance Model Questionnaire Analysis

We computed the modified TAM questionnaire factors and behavioral intention scores by summing each possible item(s) response for each factor and each participant, and then averaging the summed item response(s). The number of questions for a particular factor varied from 1 to 7 questions (Table 1). For those factors with more than 1 question, we assessed the internal consistency by calculating each factor’s Cronbach alpha. Internal consistency and other statistical testing was conducted in IBM SPSS Statistics for Windows, version 25 (IBM Corporation).

#### Qualitative Analyses

Verbatim transcripts were generated from the digital recordings and compared with the original recordings to check for accuracy prior to analysis; modifications were made as needed. The child and parent transcripts were systemically analyzed by 2 independent trained analysts using applied thematic analysis [72]. A priori structured codes, guided by the social ecological model and the theory of reasoned action/theory of planned behavior, were supplemented with emergent codes during the analyses. Coders met routinely to discuss application of the codes and emergent codes. Coding differences were discussed and resolved (JMH and AOV). All codes and definitions were recorded in a codebook maintained by the coding team. Child and parent transcripts were coded and analyzed separately. Coding was conducted using NVivo 10 for Windows (QSR International).

**Table 1:** Measured items of the modified Technology Acceptance Model theoretical factors.

Factors	Factor question(s)	Sample item
Perceived ease of use	1, 2, 3, 6, 7	Using the guided imagery mobile app appears easy to learn.
Hedonic motivation	4, 5	Using the guided imagery mobile app appears fun.
Perceived usefulness	8, 9, 10, 11, 13, 14, 16	The guided imagery mobile app appears to be useful in helping to treat abdominal pain easier.
Compatibility	12	Using the guided imagery mobile app matches well with all aspects of my everyday life.
Habit	18	I would feel comfortable when using guided imagery mobile app.
Social influence	19, 20	People who are important to me think I should use the guided imagery mobile app.
Attitude	15, 17, 24	Overall, my attitude toward the guided imagery mobile app is favorable.
Behavioral intention	21, 22, 23	I would plan to use the guided imagery mobile app frequently.

#### Integration of the Questionnaire and Interview Findings Through Triangulation

As described above, we used mixed methods (both a quantitative questionnaire and qualitative parent and child interviews) to assess acceptability, preferences, and barriers to using a GIT mobile app for treating FAPDs. As described by Creswell and Plano Clark, a mixed-methods approach is a concurrent research design that uses both qualitative and quantitative methods to explore a research question. Multiple research methods also permit comparison of the research findings through triangulation [51]. Triangulation is a process in which investigators seek convergence or collaboration between findings of various methods, including quantitative and qualitative methods [73]. We used methodological triangulation to determine whether the qualitative findings and quantitative results, when weighted equally, corroborated one another [74].

## Results

### Participant Characteristics

**Figure 2:**
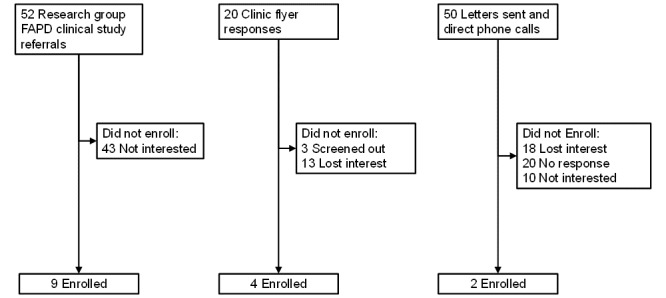
Study recruitment flow diagram. FAPD: functional abdominal pain disorder.

We enrolled a total of 15 parent-child dyads, which we deemed likely sufficient to attain theoretical saturation (ie, the point at which no new information emerges from the qualitative interviews; details outlined below) [75]. We attained sample saturation after 12 parent and child interviews; we conducted an additional 3 interviews to confirm saturation. Figure 2 outlines the outcome of our recruitment efforts.

Table 2 summarizes the child and parent demographic characteristics. All parents were mothers of the child participants. The mean (SD) maternal and child ages were 36.9 (SD 6.6) and 9.3 (SD 1.6) years, respectively. The median annual household incomes ranged from US $17,602 to US $95,137. The sample was diverse in regard to race/ethnicity, and most of the children has a diagnosis of irritable bowel syndrome (11/15, 73%; Table 2).

All mothers and most children had access to a mobile phone or electronic mobile device (Table 3). Most also had internet access through Wi-Fi or mobile cellular data. Children’s access to a computer or laptop was the same as that of their mothers, and access to a tablet among children was slightly lower than that of their mothers. More children reported using their tablets every day than a laptop or computer, or a mobile phone. Most mothers reported being comfortable using mobile phones, tablets, and laptop or desktop computers (Table 3).

**Table 2:** Child and parent demographics (N=15 mother-child dyads).

Characteristics			Sample proportion, n (%)
**Child participants**			
	**Age range (years)**		
		7-8	5 (33)
		9-10	7 (47)
		11-12	3 (20)
	**Sex**		
		Female	8 (53)
		Male	7 (47)
	**Race/ethnicity**		
		Asian non-Hispanic	1 (7)
		Black non-Hispanic	3 (20)
		White Hispanic	5 (33)
		White non-Hispanic	6 (40)
	**Functional abdominal pain disorder type**		
		Irritable bowel syndrome	11 (73)
		Functional dyspepsia	2 (13)
		Functional abdominal pain	2 (13)
**Parent participants**			
	**Age range (years)**		
		25-30	4 (27)
		31-35	2 (13)
		36-40	4 (27)
		41-45	2 (13)
		46-50	3 (20)
	**Sex**		
		Female	15 (100)
		Male	0 (0)
	**Race/ethnicity**		
		Asian non-Hispanic	1 (7)
		Black non-Hispanic	3 (20)
		White Hispanic	5 (33)
		White non-Hispanic	6 (40)
	**Median household income by zip code (US $)**		
		10,000-30,000	3 (20)
		30,001-50,000	4 (27)
		50,001-70,000	2 (13)
		70,001-90,000	4 (27)
		90,001-110,000	2 (13)

**Table 3:** Household technology ownership characteristics (N=15 mothers).

Questions	Positive responses, n (%)				
	Mobile phone	Laptop or desktop	Tablet	Wi-Fi	Mobile data
I have access to the following	14 (93)	14 (93)	15 (100)	14 (93)	12 (80)
My child has access to the following	8 (53)	14 (93)	13 (87)	13 (87)	9 (60)
I use the following on an everyday basis	14 (93)	8 (53)	10 (67)	9 (60)	10 (67)
My child uses the following on an everyday basis	4 (27)	6 (40)	11 (73)	7 (47)	4 (27)
I am comfortable with using the following	14 (93)	13 (87)	15 (100)	12 (80)	10 (67)
I feel confident when using the following	15 (100)	14 (93)	15 (100)	13 (87)	10 (67)

**Table 4:** Descriptive sample statistics of the modified Technology Acceptance Model (N=15 mothers)

Technology Acceptance Model factors	Factor response score range (minimum-maximum)^a^	Factor response score, mean (SD)^b^	Cronbach alpha
Perceived ease of use	5-25	23.1 (2.2)	.86
Hedonic motivation	2-10	7.5 (1.4)	.81
Perceived usefulness	7-35	27.5 (3.5)	.69
Compatibility	1-5	3.8 (0.8)	N/A^c^
Habit	1-5	4.5 (0.6)	N/A
Social influence	2-10	6.6 (2.0)	.84
Attitude	3-15	12.7 (1.5)	.31
Behavioral intention	3-15	12.0 (2.6)	.95

^a^Questionnaire responses were rated on a 5-point Likert scale of 1 (strongly disagree), 2 (disagree), 3 (neutral), 4 (agree), and 5 (strongly agree).

^b^Factor response scores are the average of the summed item response values for each participant.

^c^N/A: not applicable.

### Modified Technology Acceptance Model Guided Imagery Therapy Mobile App Quantitative Questionnaire

Mothers completed all questions of the modified TAM questionnaire. Table 4 summarizes the results. The Cronbach alpha of the TAM factors with more than 1 item response ranged from .31 to .95 (Table 4). The perceived usefulness (.69) and attitude factors (.31) had the lowest Cronbach alphas, which indicate a moderate and poor strength of association of the factor item responses, respectively (Table 4) [76]. Given the limited sample size and use of an adapted questionnaire, we retained all factors, including perceived usefulness and attitude, for this analysis. The mothers reported favorable behavioral intention for using a GIT mobile app to treat their affected child if such a technology were available (Table 4). In addition, all of the modified TAM factors that influence behavioral intention were highly favorable (ie, perceived ease of use, hedonic motivation, perceived usefulness, compatibility, habit, and attitude; Table 4).

### Interview Findings

#### Mothers

**Figure 3:**
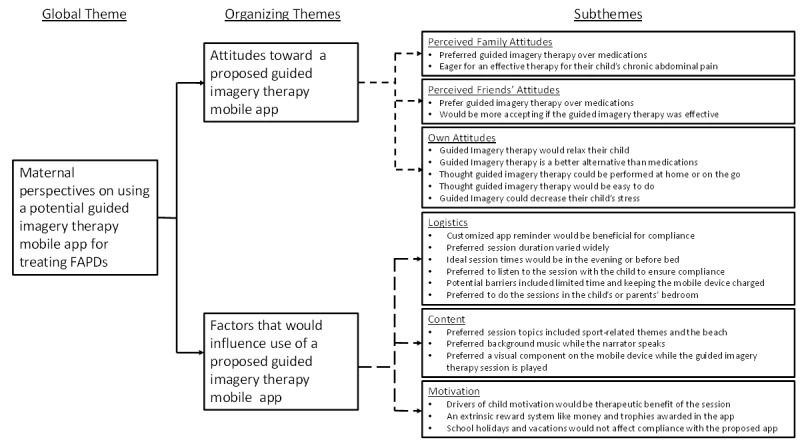
Thematic network of maternal perspectives on a proposed guided imagery therapy mobile app designed to treat functional abdominal pain disorders (FAPDs).

The maternal interviews ranged from 45 to 60 minutes. Figure 3 outlines the thematic network for the maternal interviews.

##### Attitudes Toward a Guided Imagery Therapy Mobile App

The attitudes toward the proposed app contained 3 subthemes: perceived family attitudes, perceived friends’ attitudes, and one’s own attitudes. Most mothers thought their family would be supportive of GIT, because they would prefer GIT over medications. Furthermore, they were eager to find a remedy for their child’s chronic abdominal pain.

My parents [the child’s grandparents] would be all for it, only because they hate taking medicine. They’re just like, “Oh, your medicine, in the long run, it’s gonna damage something else.” My parents would definitely be all for it [GIT sessions].

Participant 15

Mothers thought friends would be supportive of GIT and would also prefer this treatment approach over medications. In addition, mothers believed that their friends would be accepting of GIT if it improved their child’s abdominal pain.

I think they would just be really open to “Wow, you don’t have to medicate her? This will work, and you don’t have to give her medicine? This is fantastic.” I think that’s how they would go. That’s what they’d say.”

Participant 4

Mothers expressed positive self-attitudes toward the proposed app and thought the GIT sessions would relax their child. They also thought this therapy was a better option than medications to treat their child’s chronic abdominal pain. Most mothers liked the idea of having their child go to a peaceful and soothing place in their mind by listening to these sessions. Mothers also thought the GIT sessions had the potential to decrease stress for their child.

Because it’s not like experimental drugs that they would have to put in their bodies. It’s just something that they will have to listen to.

Participant 2

I’m always looking for [a] way to not use medicine or things like that [for child’s abdominal pain]. So, if it’s something that we can do, yeah I would [use GIT].

Participant 5

##### Maternal Factors Influencing Use of a Guided Imagery Therapy Mobile App

The theme of factors influencing use of a proposed GIT mobile app comprised 3 subthemes: logistics, content, and motivation.

###### Logistics

The first theme encompassed logistics for using the app. This referred to ideal characteristics and conditions for its use. Mothers thought reminders to encourage their child to use the app would be helpful; however, there were varying opinions on the format for the reminders (eg, text messages, emails). There were also varying suggestions for the ideal time to receive a reminder. A customizable reminder option in which they could set a reminder alarm was suggested. Despite the customization of the reminder, the mothers thought a once-a-day reminder would be sufficient.

I think it [an app reminder] would be very helpful because you get—I tend to forget homework, band, and everything that goes into your daily activities or your daily life is just—I would definitely say a reminder would be very helpful.

Participant 14

In regard to the timing of the sessions, the mothers thought the evening or before bed would be the ideal time to listen to GIT sessions. They also stated that their child would listen to the session in their own or the parent’s bedroom. The suggested session duration was no longer than 15 minutes due to concerns that the child may not be able to pay attention for a longer duration. The mothers also preferred to listen to the sessions with the child to ensure therapeutic compliance.

But in the morning, I feel like they have enough trouble getting up, getting themselves ready to go, so maybe not necessarily in the morning.

Participant 10

Because I think that if it [GIT sessions] went a lot longer than [15 minutes] she would get bored and she wouldn’t want to do it anymore.

Participant 2

The mothers identified potential problems that could arise during use of the app. They were concerned that their busy lifestyle could interfere with listening to the sessions consistently. The mothers also were concerned that the child’s mobile device may not be charged as needed to conduct the sessions.

To really stay on it [listening to the GIT sessions], to do it, to make sure we’re doing it. Just ’cause the day is wacky busy sometimes, and things happen. You’re not at home when you thought you were gonna be, or the morning just gets crazy, and you don’t have that time to pull away.

Participant 4

###### Content of Guided Imagery Therapy Sessions

We defined the content theme as opinions about the ideal information to include or not include in GIT sessions. The mothers thought that sports-related themes such as soccer and a beach location would be ideal topics for the GIT sessions. They also stated that they would prefer background music to go along with the narration. They also thought that a visual image on the mobile device would be ideal to accompany the GIT sessions. The recommended on-screen visuals varied among the mothers but ranged from a static related picture of the concurrent GIT session to changing colors and mazes.

He likes basketball, soccer. Imagine if you are playing soccer and you’re in a soccer field and you don’t do this and you don’t do this, or you do this, your stomach pain is not gonna come, something like that, or how well you should exercise or bicycle and it can cure your stomach pain, and it will make your legs feel better. These things are the activities that these kids do.

Participant 13

So, I’d like soothing music. So there’s no background. And so if we’re trying to relax, often times, and kids, often times, they play soothing music in the background. I mean what are some of the things you do to get a baby to sleep? Sometimes they have the light noise, the soothing backgrounds.

Participant 9

Maybe have a picture that they can see if they open their eyes and they can see a picture of a swing or someone swinging, just to help them out a little bit. Because like I said, I’m a visual person. I like to see things so that helps me comprehend a little bit more.

Participant 5

###### Motivation

The final theme for the maternal interviews was motivation. We defined this theme as mothers’ perspectives on factors that may affect their child’s willingness to use the app. The mothers thought their child would be motivated to listen to the sessions if the sessions led to therapeutic benefit for their FAPD-related abdominal pain. They also thought that a reward system based on compliance using the GIT sessions would be ideal. Their responses about specific types of rewards varied but included candy, money, a game on the mobile device, and even trophies within the proposed app. Mothers thought their child’s motivation to use such a mobile app would not change during holiday breaks or summer vacations.

I know that if it [the proposed GIT mobile app] really does get rid of her abdominal pain, she would do it. She’s to the point where if it worked, she would do it. I think that if it worked, I wouldn’t have to say today we’re gonna do—she would remind me. “Mom, we didn’t do what we were supposed to do.” She would be motivated, I think, to do it, if it really did work for her.

Participant 4

But yeah, I mean he does school, they’re not apps, but they’re school websites that you go to where you actually have little math sessions. And when you complete a certain level of math session, then you get a trophy or get a star or whatever it is. And you elevate to different levels, that kind of thing.

Participant 7

#### Children

**Figure 4:**
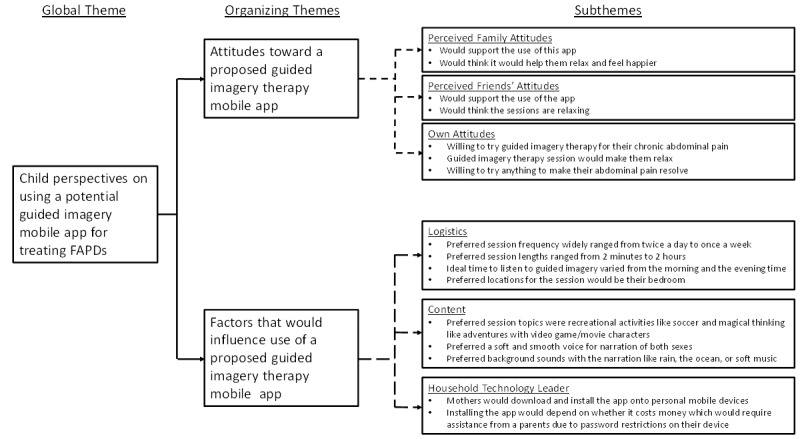
Thematic network of child perspectives on a guided imagery therapy mobile app designed to treat functional abdominal pain disorders (FAPDs).

The child interviews lasted from 15 to 40 minutes. Figure 4 outlines the thematic network for the child interviews.

##### Attitudes Toward a Guided Imagery Therapy Mobile App

The attitudes theme reflected thoughts on a GIT mobile app that treats abdominal pain related to their FAPD. Similar to the previous analysis, this theme had 3 subthemes: perceived family, perceived friends’, and one’s own attitudes. The children thought their family would support listening to these sessions as treatment for their abdominal pain. The children also thought that their friends would either be indifferent to or supportive of their using such an app. In regard to self-attitude toward the proposed app, the children liked the GIT session excerpt and wanted to try out these sessions to help with their abdominal pain. They thought the session excerpt was relaxing and these sessions would change their mood in a positive sense.

Well, they’d [participant’s family would] be fine because it’s trying to help me feel better.

Participant 7

Because I don’t know it [guided imagery therapy session excerpt] makes me feel—because most of the time I get dizzy and stuff because I’m very stressed out and I just don’t wanna have to think about school and everything I have to do. And I will try this out because, I don’t know, it would get those things off my mind at the moment so I could just kind of—I don’t know like relax.

Participant 6

##### Factors Influencing Use of a Guided Imagery Therapy Mobile App

This theme reflected how the children would use the app. Subthemes that emerged were logistics, content, and household technology leader.

###### Logistics

Logistics referred to ideal characteristics and conditions for using a GIT app. The children had no consensus on the suggested duration or frequency of a GIT session. The duration and frequency responses ranged from 2 minutes to 2 hours and once a week to twice daily, respectively. The ideal time to listen to the sessions would be in the morning or evening. Affected children stated that they would prefer to listen to the sessions in their bedroom.

Because when you wake up because your scared. What if you’re going to school, if there’s gonna be a test and your stomach might hurt in the mornings.

Participant 4

Up in my bedroom. You literally can’t hear anyone downstairs besides if they shout up.

Participant 3

###### Content of Guided Imagery Therapy Sessions

We defined the content theme as opinions about the ideal information to include or not include in guided imagery sessions for a mobile app. The child participants stated that they would prefer to listen to guided imagery sessions focused on various recreational or sporting activities and magical thinking. These session topics included sports themes (eg, lacrosse and soccer), animals, video game characters and related adventures, and food. A soft and smooth voice for GIT narration was preferred. They also preferred background sounds with the session narration. The children liked background sounds such as rain, the ocean, and soft music.

I’d really like to hear imagine you’re in a place with a bunch of kitty cats. And then they would have kitty cat sounds in the background…

Participant 12

I love lacrosse, so I scored the winning goal or something and that would probably help me.

Participant 8

###### Household Technology Leader

We defined this theme as the persons responsible for downloading and installing the app onto household electronic mobile devices. Children stated that their parent would be the person to download and install the app. They also stated that downloading the app to their device would depend on whether the app costs money and, if so, this process would definitely require a parent to complete the app installation.

#### Triangulation

Acceptability of the proposed GIT mobile app was addressed in both the quantitative questionnaire and qualitative interviews. The questionnaire demonstrated a favorable behavioral intention score to use the app and attitude score toward the app. The interviews also addressed this issue through the positive maternal and child attitude themes. In this case, the questionnaire results were corroborated by the qualitative rich data. Both mothers and affected children supported the app because they did not like giving or taking medications for FAPD-related abdominal pain, they were eager for another modality for treatment, and the sessions could make affected children feel good.

The concept of motivation was addressed in both quantitative and qualitative methods. Hedonic motivation examines the intrinsic motivation to use a technology. The maternal and child interviews both addressed intrinsic motivation as a subtheme in the theme of influencing factors. Integration of these methods findings also showed corroboration between the methods and further insight into why there was a favorable hedonic motivation score in the questionnaire. The mothers insisted that the therapeutic benefit of such an app would drive their affected children to use the app. These data suggest that the mothers expected a GIT mobile device to be therapeutic if it were available.

Perceived ease of use seemed to be congruent between both the quantitative questionnaire and the qualitative interviews. The TAM questionnaire revealed a favorable perceived ease-of-use score regarding the proposed app. Parental interviews supported this same finding because the parents were familiar with reminder alerts on mobile devices and text messages. Furthermore, the child interviews mentioned that their parents would be responsible for manipulating their mobile device to install such an app. The mothers and their children were familiar with mobile devices; hence, perceived ease of use of a proposed GIT mobile app is plausible.

## Discussion

The use of psychological therapies for FAPDs is recommended by experts, as they have been shown to be effective [15,77]. Unfortunately, accessing such therapies is problematic due to limited access to trained specialists and restricted insurance coverage for such therapies [26,27]. The utility of remotely delivered psychological therapies for FAPDs (eg, delivered by compact disc or telephone) appears highly promising [26,28-30,78]. However, these methods also have drawbacks (eg, compact disc use is unpopular). As a solution, the ubiquity of personal electronic mobile devices could permit low-cost, large-scale distribution of prerecorded audio GIT sessions to children with FAPDs.

### Principal Findings

This study is the first, to our knowledge, to examine the perspectives of children with FAPDs and their parents on a proposed mobile app to remotely deliver GIT sessions. We successfully recruited and captured the thoughts of a diverse representative patient sample and their mothers, and they both demonstrated support for this technology-based treatment approach through a quantitative questionnaire and qualitative interviews. Our modified TAM questionnaire was completed by affected children’s mothers, and this instrument discovered favorable behavioral intention to use such a device, along with other factors that affect use of health technology. The maternal and child interviews confirmed their interest and provided explicit detail for supporting such a technology. Furthermore, the participants provided key information about their preferences for the app and how they would use the health technology tool in their everyday lives. Integration of our quantitative questionnaire and qualitative interview findings also demonstrated favorable motivation and perceived ease of use for such an app.

The study questionnaire affirmed that affected children and their mothers typically had access to electronic mobile devices and internet access, and this finding aligns with other US national studies [79,80]. Beyond possession of these technologies, mothers and their children exhibited features of proficiency with these devices, as they were familiar with app reminders, text messages, and calendar reminders on their mobile devices. Our results align with a previous study reporting that parents saw value in a reminder feature for medication compliance [56]. The children also confirmed that they would have to give their mobile device to their parent download and install apps. This patient population and their families appeared to have the required hardware and technical proficiency to use a GIT mobile app.

The qualitative analyses of the maternal and child interviews had similar organizing themes of attitudes toward a proposed GIT app and factors that would affect use of such an app. This is likely an artifact of the purpose of the study (to determine acceptability and appropriateness of using a GIT to treat FAPDs) and closely related maternal and child interview scripts. Despite the similarities in the maternal and child thematic networks, the mothers and children provided unique responses (eg, mothers thought a beach location would be ideal for GIT session topic content, but the children endorsed other topics such as animals, adventures, and food as topic content of interest). Such differences will need to be further explored during the app development phase in the future.

Our mixed-methods approach confirmed acceptability of a proposed GIT mobile app to treat FAPDs in children. Both mothers and their affected children did not like giving or taking medications, especially when they did not seem to help the children’s abdominal pain. We suspect that parents are willing to accept this technology because parents already feel comfortable using electronic mobile devices to calm and distract young children [81]. Our findings are congruent with a qualitative focus group study of adolescents with chronic pain who confirmed the utility of various methods to treat their pain, including psychological approaches [82]. Our results suggest that external factors, such as family members and friends, do not seem to be a barrier to using this proposed clinical tool.

Motivation to use the app appeared favorable among affected children and their parents. the intrinsic motivation of the app’s therapeutic effects would drive its use. The study also identified extrinsic motivation through external rewards such as money and app-based trophies. Further studies are needed to evaluate the value of intrinsic benefit versus extrinsic reward and how long extrinsic awards should be maintained or even if they should be started.

There is a growing body of literature that supports a user-centered approach for mobile health app development, and our study findings suggest why this is important. Our qualitative interviews revealed important features that will need to be incorporated in app development to increase therapeutic compliance among pediatric patients (eg, specific GIT session topics and app reminders). Zhao et al suggested adding features to mobile health apps to improve their effectiveness, including those that offer less time consumption, user-friendly design, real-time feedback, individualized elements, detailed information, and health professional involvement [83]. Incorporating these key study results should optimize compliance and encourage pediatric patient motivation.

### Limitations

The generalizability of this study is restricted, as we characterized 15 mother-child dyads for our study’s conclusion. However, the demographics of our sample aligned with the patient population within our pediatric health care system, which is in the most racially and ethnically diverse large city in the United States. In addition, we used thematic saturation to minimize bias. Our limited study sample did not allow us to determine which specific factors of the modified TAM or other unforeseen factors were most influential in predicting behavioral intention for using the proposed GIT mobile app.

### Conclusion

Based on the results of our study, a GIT mobile app designed to treat pediatric patients with FAPDs has the potential to be a well-received, evidence-based clinical tool similar to other mobile apps designed to treat anxiety or depression [36,37,84]. This study identified key features to incorporate into the design of the mobile app (eg, reminder features, ideal session topics, and background music in addition to narration) to increase compliance. These findings provide a strong foundation to develop and deploy a GIT mobile app into clinical practice and establish a new paradigm for treating FAPDs in the pediatric population.

## Appendix

Multimedia Appendix 1 Modified Technology Acceptance Model questionnaire for a guided imagery mobile app.

Multimedia Appendix 2 Parent interview script.

Multimedia Appendix 3 Child interview script.

## Abbreviations

FAPD: functional abdominal pain disorder
GIT: guided imagery therapy
TAM: Technology Acceptance Model
