# Supplementary material for: Maternal and Child Acceptability of a Proposed Guided Imagery Therapy Mobile App Designed to Treat Functional Abdominal Pain Disorders in Children: Mixed-Methods Predevelopment Formative Research
Source: JMIR Pediatr Parent. 2018 Jun 29;1(1):e6. doi: 10.2196/pediatrics.8535 (PMC6716440; doi:10.2196/pediatrics.8535)
Supplement: Multimedia Appendix 1 [file pediatrics_v1i1e6_app1.pdf]

Appendix A

Modified Technology Acceptance Model Questionnaire for a Guided Imagery Mobile Application

We are interested in using guided imagery therapy sessions delivered through a mobile app to treat children with chronic abdominal pain. Please complete the following questionnaire.

		Strongly Disagree	Disagree	Neutral	Agree	Strongly Agree
1	The guided imagery mobile app appears easy to use.					
2	Using the guided imagery mobile app appears clear and understandable.					
3	Using the guided imagery mobile app appears easy to learn.					
4	Using the guided imagery mobile app appears fun.					
5	Using the guided imagery app appears enjoyable.					
6	I would be able to navigate through the guided imagery mobile app.					
7	I feel I would have control over the use of the guided imagery mobile app.					
8	The guided imagery mobile app would fit in well with my day to day activities.					
9	The guided imagery mobile app					

	appears useful in helping to treat abdominal pain.					
10	The guided imagery mobile app appears to be useful in helping to treat abdominal pain easier.					
11	The guided imagery mobile app would help me to treat abdominal pain more quickly.					
12	Using the guided imagery mobile app matches well with all aspects of my everyday life.					
13	The guided imagery mobile app would help me treat abdominal pain so that I can take part in other activities.					
14	Some mobile apps can help me solve problems.					
15	I would feel good if the guided imagery mobile app helped me figure out to treat abdominal pain.					
16	Using the features of the guided imagery mobile app appears easy to remember.					
17	I would like using the guided imagery mobile app.					
18	I would feel comfortable when using guided imagery mobile app.					
19	People who are important to me think I should use the guided imagery mobile app.					
2	People whose opinions that I value					

0	prefer that I use the guided imagery mobile app.					
21	I would use the guided imagery mobile app in the future					
22	I would always try to use the guided imagery mobile app for abdominal pain.					
23	I would plan to use the guided imagery mobile app frequently.					
24	Overall, my attitude towards the guided imagery mobile app is favorable.					
