# Supplementary material for: Maternal and Child Acceptability of a Proposed Guided Imagery Therapy Mobile App Designed to Treat Functional Abdominal Pain Disorders in Children: Mixed-Methods Predevelopment Formative Research
Source: JMIR Pediatr Parent. 2018 Jun 29;1(1):e6. doi: 10.2196/pediatrics.8535 (PMC6716440; doi:10.2196/pediatrics.8535)
Supplement: Multimedia Appendix 2 [file pediatrics_v1i1e6_app2.pdf]

Appendix B

Parent Interview Script

Thank you for participating in our research study.  Today I would like to ask you a series of questions about your child’s belly pain and a potential treatment option.  Please know that there are no right or wrong answers to these questions.  Please share your honest thoughts and opinions regarding these topics.  We are recording this session so we can study your thoughts on how we can best help children for belly pain.

We are in the process of developing a new therapy for abdominal pain that is not a medication.  It requires a child to listen to pre-recorded audio sessions that will help them relax and take control of their belly pain. We also encourage the parents to listen to the sessions with their child.  The therapy would be accessed through a mobile app (display example of app) typically found on electronic devices, like a smartphone or computer. It is called guided imagery.  To use the app, the child would need to mail.be in a quiet environment when they listen to these pre-recorded sessions.  Here’s an example of what a session would sound like.  <Play 2 minute clip of guided imagery session excerpt>

1. What do you think about the guided imagery session you just heard?

2. How would you change or improve the guided imagery session you just heard?
    a. (Prompt): What are some things you would add or remove from the guided imagery sessions?

3. Now that you have heard a guided imagery session clip, do you think this type of treatment could help your child’s abdominal pain?

a. Please help me understand your answer.

b. Probe to determine if it's something they would be willing to try and why/why not.

4. We envision parents doing the sessions with their child. What do you think about this?

5. Where could you and your child engage in listening to the guided imagery sessions?
   a. (Prompt): Before school, after school, or before bed.
6. Where could you and your child engage in listening to the guided imagery sessions?

7. What would your family think about your child using this recorded guided imagery therapy to help their belly pain? What about your friends?

8. Recorded guided imagery requires patients to use the sessions almost every day to be effective for about eight weeks. What do you think about receiving daily reminders that encourage you to listen to the guided imagery session?
   a. How often should the reminders be sent? What type of information would you like conveyed in the reminders?

9. How long should each session be? Probe 15 min, 25 min

10. On a scale of 1-3 (with 3 having the highest motivation), how motivated would your child be to listen to the sessions? Please help me understand your reason for selecting this answer.

    a. (Prompt): What are some things that motivate your child?

    b. (Prompt): Does motivation change during breaks from school (e.g. summer or winter break)?

11. To listen to the sessions, you and your child would need to be in a quiet place with few distractions. Where would this be?

12. What rules, if any, does your family have about cell phone or computer use that may prevent you and your child from using the mobile app?

13. Does your child ever go over their allotted cellphone internet usage?  If so, how often?
14. If the recorded guided imagery sessions were to be delivered through a mobile phone and your child has to use your phone for these sessions, would you be willing to block phone calls, texts, and other notifications during your child's guided imagery sessions daily for 15-25 minutes?
15. What challenges do you think you could face using the guided imagery sessions delivered by a mobile app?

This is the conclusion of the interview.  Thanks for providing me with your thoughts regarding these topics.  The information provided will be taken into consideration as I try to find the best way to help kids with abdominal pain.  At this time, I would like to compensate you $20 cash for your participation in completing this interview and additional survey.  I will also validate your parking today.  Thanks for your participation and have a great day.
