# Supplementary material for: Maternal and Child Acceptability of a Proposed Guided Imagery Therapy Mobile App Designed to Treat Functional Abdominal Pain Disorders in Children: Mixed-Methods Predevelopment Formative Research
Source: JMIR Pediatr Parent. 2018 Jun 29;1(1):e6. doi: 10.2196/pediatrics.8535 (PMC6716440; doi:10.2196/pediatrics.8535)
Supplement: Multimedia Appendix 3 [file pediatrics_v1i1e6_app3.pdf]

Appendix C

Child Interview Script

Thank you for talking with me. Today I will ask you some questions about your belly pain and something that might make it better. I am asking you these questions because you are the expert and can tell me what others your age think. So, please be sure to tell me what you really think – you are the expert, and I really need your help! There are no right or wrong answers – just your thoughts and opinions. We will record this session so we can study your answers.

We are developing a new way to deal with belly pain. It's not a medicine. Instead, you would listen to special recordings that help you relax which might make your belly hurt less.

1. What are some things that help you relax? (Prompts: soft music (what kind), sounds of rain, ocean, etc.)

The recordings would be delivered through an app <show example of mobile app>. An example of a session would sound like this. <Play 2 minute audio excerpt of guided imagery session>.

2. What do you think about this? Probe to determine if they would be willing to try the treatment and why/why not
3. What do you think family and friends would think?
4. On a scale of 1 to 3 with 3 being the most helpful, do you think listening to the sessions could make your belly pain better?
    a. Could you help me understand your answer?

5. How did the person talking on the recording sound to you? Probe to identify likes/dislikes and how voice make them feel.
6. What are some things you would like to hear using this type of treatment?
    a. Would sounds like rain or the ocean be helpful? Would anything else be helpful for relaxing?
7. How often do you think parents and children should listen to these recordings? Probe to identify their reasons for saying this; also probe to identify their thoughts about daily sessions vs just when their belly hurts.
8. How long should each session last?
9. When is the best time of day to listen to these recordings?
    a. (Prompt): Maybe before school, after school, or before bed.
10. Where would you listen to the recordings? Probe to identify locations; if more than one mentioned, probe to identify most likely place.
11. To listen to the recordings, you and your parent would need to download an app to a mobile device or computer. What do you think about this? Probe to identify who would be most likely to do this (parent, child, both).

Before we end, is there anything you want to add or say?

That's all of my questions. Thank you for talking with me today. Do you have any questions for me? Thanks for providing me with your thoughts regarding these topics. At this time, I would like to bring your parent back into the room so we can wrap up this session.
